# A fresh look at sports PSM-systems

**DOI:** 10.2478/joeb-2023-0003

**Published:** 2023-05-26

**Authors:** Vladimir Savostyanov, Alexander Kobelev, Anton Govorin

**Affiliations:** 1. Faculty of Biomedical Engineering, Bauman Moscow State Technical University, Moscow, Russia

**Keywords:** bioimpedance, rheography, sport, heart rate, respiratory rate, monitoring, functional athlete readiness

## Abstract

The aim of the proposed study is to reveal the correlations between the dynamics of Respiratory Rate (RR) and Heart Rate (HR) during intermittent physical work at maximum power on a cycle ergometer. The stage of investigating the General functional athlete readiness (GFAR) was conducted using the sports standard “R-Engine” and the cycle ergometer in 16 volunteers (10 men, 6 women) whose average age was 21±1.17 years. To determine the athletic potential of the volunteers in this study, we used our own Coefficient of Anaerobic Capacity (CANAC Q, beats). Continuous registration of the heart rate and respiratory rate of volunteers in the maximum power sports test was performed by the “RheoCardioMonitor” system with a module of the athlete functional readiness based on the method of Transthoracic electrical impedance rheography (TEIRG). The degree of correlation of functional indicators (M, HRM, GFAR) with CANAC Q in all experimental series of the study group as a whole (n=80) was at a very high level, which confirmed the effectiveness of using the Coefficient of Anaerobic Capacity (CANAC Q) in assessing the general functional athlete readiness of the volunteers. CANAC Q is measured in “beats” of the heart and is recorded very accurately using the method of transthoracic electrical impedance rheography (TEIRG). For this reason, as a promising sports PSM-system, CANAC Q can replace the methods for determining the functional athlete readiness by blood lactate concentration and maximum oxygen consumption.

## Introduction

Physiological state monitoring systems (PSM-systems) are IT systems used to record various physiological parameters of the human body [1]. The purpose of the PSM-systems is to monitor the viability of a person in extreme conditions (diseases, injuries, extreme physical exertion, etc.) [2].

It is generally accepted that the degree of human efficiency in conditions of high intensity and tension (sports of high achievements, extreme activities, etc.) is determined by a complex interaction of the following components: 1) Health condition; 2) Physical (athletic) condition; 3) Technical (special) education; 4) Psychological motivation (moral and volitional qualities).

For a clear understanding of the interaction of these components in Sport Pedagogy, the term “Functional Athlete Readiness” is used [3]. The concept of “Functional athlete readiness” (FAR) has a very complex and multifaceted context [4].

The FAR may be defined as a relatively stable state of the organism, determined by the level of development of key functions required for a particular sport, as well as their specialized properties that directly or indirectly determine the effectiveness of the competitive activity [5].

The FAR is divided into two types:

a) General functional athlete readiness (GFAR), which characterizes the general physical development and endurance.
b) Special functional athlete readiness (SFAR) depending on the type of sport.

## Research methods and objects

During 2015-2017 the Bauman Moscow State Technical University together with The University of Alabama (USA) carried out a scientific project “The study of applicability of the breathing sensor PACT2.0 in determining aerobic-anaerobic potential of professional athletes (junior ice hockey players)”. This study was approved by the Institutional Review Board for the Protection of Human Subjects request for approval of research involving human subjects (IRB Protocol #15-024-ME 2015/12/14), as well as by the Decisions of the Ethics Committee of Bauman Moscow State Technical University № 1 from 2015/11/11 and № 2 from 2016/10/01).

The purpose of this study was to identify correlations between the dynamics of Respiratory Rate (RR) and Heart Rate (HR) during intermittent work at maximum power on a cycle ergometer. The study allowed to determine anaerobic capacity of sportsmen in the framework of their general functional athlete readiness and to evaluate the feasibility of developing a sports methodology for diagnostics of chronic overstrain syndrome of the cardiovascular system.

The necessity for this research is due to the current requirements of the strategical point of a coach to the information about how professional athletes in team sports (hockey, football, handball, basketball, etc.) correspond to each other in speed and strength qualities with the definition of the possibility of their mutual substitution without loss of game quality in the competitive cycles.

The original PSM-system developed during this study was called the sports standard “R-Engine”. The “R-Engine” solves the global problem of determining the individual dynamics of changes in the levels of functional readiness of each member of the sports team, which allows to effectively maintain a high level of performance of the team as a whole during a competitive macrocycle.

The convenience of this sports functional testing is that it is performed directly in the stadium conditions without involving any complex diagnostic medical equipment. The heart rate (HR) is most often used as a criterion for assessing the intensity of exercise in sport. There is a linear relationship between heart rate and training intensity [6].

Endurance training (aerobic exercises) is often performed by athletes at a heart rate of about 180 beats per minute (bpm). However, for many athletes this heart rate significantly exceeds their personal aerobic-anaerobic transition area.

Therefore, Resting heart rate (HR0), Maximum heart rate (HRmax), Reserve heart rate (HRR), and Target heart rate for the transition to the fully anaerobic zone (HRM) should be used to calculate the athlete’s training intensity and to monitor their functional status. HR_max_ is the maximum number of contractions that the heart can make within one minute. After the age of twenty, HR_max_ gradually begins to decrease by about one beat per year. So HR_max_ is calculated using the following formula [7]:

1 $$HR_{max}=220-Age\left(years\right)$$

The Heart Rate Reserve (HRR) Method, which was developed by the Finnish scientist Karvonen, is also used to calculate exercise intensity. HRR is the difference between HRmax and resting HR (HR0):

2 $$HR_{r}=HR_{max}-HR_{0}$$

Knowing the HR_R_, you can calculate the target of the heart rate (HR_M_). Target heart rate (HR_M_) is the optimal heart rate, characterizing the transition to the clear anaerobic zone at an exercise intensity of M (%):

3 $$HR_{M}=HR_{0}+M\times HR_{R}$$

At the same time, knowing HR0 and HRmax, you can use the Karvonen formula to calculate at what intensity (M) the athlete performs the exercise:

4 $$M=\frac{\left(HR_{diring\;exercise}-HR_{0}\right)}{\left(HR_{max}-HR_{0}\right)}\times 100\%$$

Thus,

5 $$M=\frac{\left(HR_{diring\;exercise}-HR_{0}\right)}{\left(220-Age-HR_{0}\right)}\times 100\%$$

The sports standard “R-Engine” was created for functional testing of professional athletes in training camps and competitions. This functional testing is based on registration of various biometric and anthropometric indicators with subsequent complex mathematical processing.

**Figure 1.**
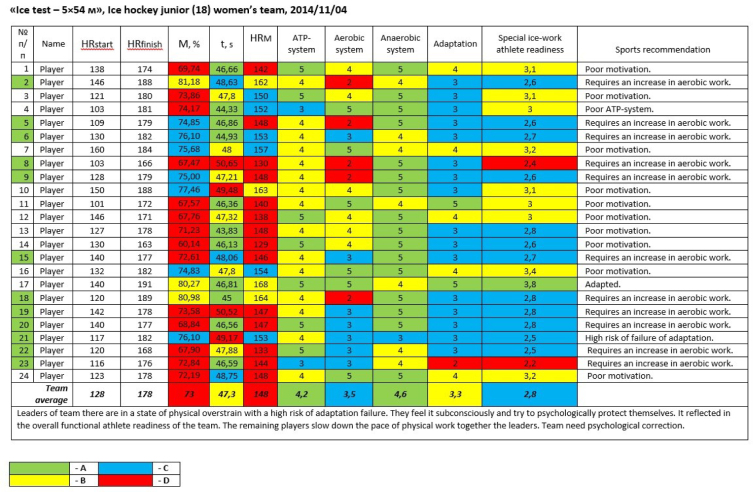
Pedagogical interpretation of the sports standard “R-Engine” results.

Based on the results of the “R-Engine” sports standard, an individual medical-biological program is made including recommendations (Figure 1):

1 Additional work or rest at educational training camps;
2 Preventive measures;
3 Use of selective methods of restoring the functions of ATP-system, Aerobic- and Anaerobic-system;
4 Plan of correction of medical and biological support and pharmacological protection in extreme conditions of sport.

The created sports standard “R-Engine” proved to be very effective in the training of professional athletes. Validation of the “R-Engine” served as methods for determining the athlete functional readiness by blood lactate concentration and maximum oxygen consumption.

However, it requires the use of special software, which is not available to a wide audience of users. For this reason, we decided to simplify this method by using the diagnostic variant of studying only the General functional athlete readiness.

The stage of the study of the General functional athlete readiness (GFAR) was conducted using the sports standard “R-Engine” and the cycle ergometer in 16 volunteers (10 men, 6 women) whose average age was 21±1.17 years. All volunteers were members of the sports club of our university.

The use of the test was based on the athletes achieving maximum power of muscular load in five experimental series of 45 sec each, in which the heart rate rises to maximum values, followed by recovery pauses of 90 sec each.

To determine the sports potential of the volunteers in this study, we used our own Coefficient of Anaerobic Capacity (CANAC Q, beats):

6 $$Q=\sqrt{\frac{HR_{start}}{RR_{start}}.\frac{HR_{finish}}{RR_{finish}}}$$

Continuous registration of the heart rate and respiratory rate of volunteers in the maximum power sports test was performed by the “RheoCardioMonitor” system with a module of the athlete functional readiness based on the method of Transthoracic electrical impedance rheography [8-12] (TEIRG).

The method of Transthoracic electrical impedance rheography allows to register frequency characteristics of bioimpedance response of a volunteer to the sport test of maximum power, thus simultaneously registering the characteristics of his productive work of the heart channel, efficiency of gas transport function of blood, and also reliably estimating his reactivity and adaptive potential [13].

In technical terms, the “ReoCardioMonitor” system represents a two-channel impedance-measuring converter. The first channel is designed to measure the transthoracic impedance of the chest (base and pulse components, impedance-breathing pattern) with an ECG measurement channel from the same impedance electrodes. The transthoracic channel is used to calculate stroke volume and cardiac output. The second channel measures breathing pattern [14].

**Figure 2.**
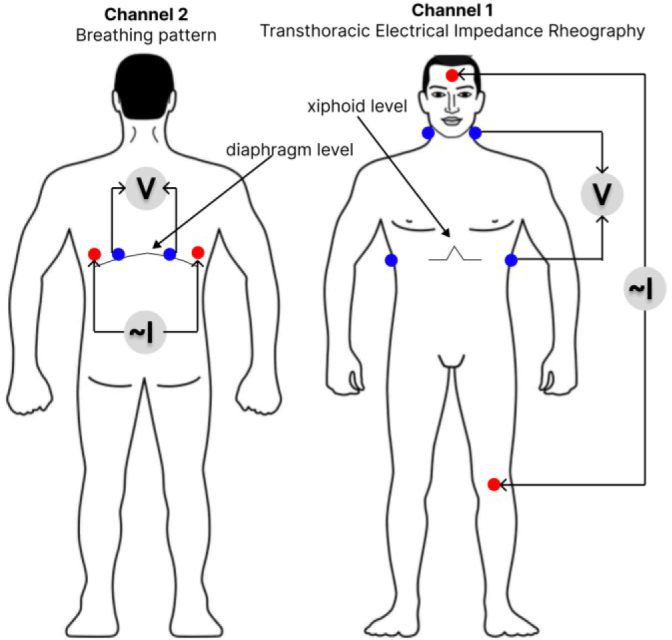
Electrode application diagram.

The measurement method is tetrapolar. A source generating alternating current of high frequency is connected to the current electrodes. The signal is recorded from the potential electrodes. As the distance between the current and potential electrodes increases, their influence on each other decreases and the accuracy of the measurement result increases [13]. Figure 2 shows the selected electrode application scheme.

Using such a scheme of applying the electrodes ensures greater uniformity of current flow through the studied area and reduces the influence of resistance variations at the electrode-skin interface on the measurement result [14].

Contact with the patient’s body was provided by the use of White Sensor 4500 disposable ECG electrodes from the “Ambu” company, electrode size 50×48 mm. The internal electrodes, marked in blue in the diagram, measure and register the voltage change ΔU(t), which is used to calculate and register the impedance signal dZ. External electrodes should carry an alternating current with a high frequency of 100 kHz at a low amplitude of 8 mA. The impedance change is recorded as a function of time Z(t).

### Confirmation of Ethical Compliance

This study was approved by the Institutional Review Board for the Protection of Human Subjects request for approval of research involving human subjects (IRB Protocol #15-024-ME 2015/12/14), as well as by the Decisions of the Ethics Committee of Bauman Moscow State Technical University № 1 № 1 from 2015/11/11 and № 2 from 2016/10/01). Informed consent was obtained from all subjects participating in the study. Written informed consent for the publication of this work was obtained from the 16 volunteers.

## Results and discussion

The classification of the studied functional indicators are shown in Table 1.

**Table 1.** Classification of the studied functional indicators.

Indicators	Result	Score
The intensity of the performed exercise (M, %) *	≥ 85	A
	80-84.9	B
	75-79.9	C
	≤ 74.9	D
Target heart rate for the transition to completely anaerobic zone (HRM, bpm) *	≥ 171	A
	161-170	B
	151-160	C
	≤ 150	D
General functional athlete readiness (GFAR, units) *	≥ 3.5	A
	3.0-3.4	B
	2.5-2.9	C
	≤ 2.4	D
Coefficient of Anaerobic Capacity (CANAC Q, beats)	≥ 10.1	A
	7.5-10	B
	5.5-7.4	C
	≤ 5.4	D

1 * - classification of Russian Ice Hockey Federation [4].

The results of the study (Table 2) indicate a low level of the General Functional Athlete Readiness of volunteers: on average GFAR=2.5 units (Score “C”) were in all test series. Professional athletes should have a GFAR of at least 3.5 units.

**Table 2.** Results of the study of the General Functional Athlete Readiness with using the method "R-Engine" in all experimental series separately.

Indicators	1st series	2nd series	3rd series	4th series	5th series
HR_start_, bpm	102.8 ± 13.6	137.1 ± 15.7	144.4 ± 17.9	148.2 ± 15.1	152.1 ± 16.2
HR_finish_, bpm	158.4 ± 15.7	167.9 ± 14.7	173.1 ± 11.9	173.9 ± 11.6	176.8 ± 11.9
RR_start_, rpm	17.1 ± 6.9	19.4 ± 4.7	22.1 ± 5.7	23.4 ± 4.7	24.1 ± 6.0
RR_finish_, rpm	33.7 ± 6.5	35.1 ± 5.1	37.6 ± 7.2	39.5 ± 7.9	40.0 ± 7.6
M, %	66.7 ± 8.4	65.9 ± 7.2	67.7 ± 5.9	67.2 ± 7.1	68.4 ± 7.0
HRM, bpm	129.5 ± 18.1	134.7 ± 17.4	134.7 ± 17.4	140.1 ± 15.7	143.4 ± 15.7
GFAR, units	2.51 ± 0.70	2.48 ± 0.63	2.59 ± 0.64	2.54 ± 0.60	2.61 ± 0.57
CANAC Q, beats	5.7 ± 1.29	5.9 ± 0.87	5.7 ± 1.13	5.4 ± 0.92	5.5 ± 0.99

The value of individual exercise intensity (power) (M) did not reach 70% (Score “D”) in any of the test series. The threshold value of anaerobic metabolic rate (HRM) does not go beyond 145 bpm (Score “D”). The level of professional athletic performance must be at least 171 bpm.

Positive correlations of functional indicators (M, HRM, GFAR) with the Coefficient of Anaerobic Capacity (CANAC Q) were registered in the first two test series. However, when the series of work with increased power continued, the correlations between the functional indicators began to break down due to pronounced fatigue of the volunteers (Table 3).

**Table 3.** The degree of correlation of functional indicators with CANAC Q in the study.

Indicators	1st series	2nd series	3rd series	4th series	5th series
M, %	r = 0.50	r = 0.41	r = 0.18	r = 0.09	r = 0.02
	p < 0.01	p < 0.01	p > 0.05	p > 0.05	p > 0.05
HRM, bpm	r = 0.55	r = 0.40	r = 0.19	r = 0.08	r = 0.04
	p < 0.01	p < 0.01	p > 0.05	p > 0.05	p > 0.05
GFAR, units	r = 0.53	r = 0.51	r = 0.38	r = 0.29	r = 0.33
	p < 0.01	p < 0.01	p < 0.05	p > 0.05	p > 0.05

The average maximum heart rate (HR_finish_) of volunteers in all experimental series of the study group as a whole (n=80) failed to pass the 170.0±14.8 bpm mark, although according to the Karvoner’s formula, their maximum heart rate should reach 199 bpm. The heart rate start (HR_start_) was 136.9± 23.7 bpm. The maximum respiratory rate (RR_finish_) also turned out to be excessively high 37.1±7.4 rpm. At the same time the respiratory rate start (RR_start_) was 21.2 ± 6.2 rpm, which also indicates a very poor physical performance of this group of volunteers (Table 4).

**Table 4.** Classification of the studied functional indicators.

Functional indicators	Group in full (n=80)
HR_start_, bpm	136.9 ± 23.7
HR_finish_, bpm	170.0 ± 14.8
RR_start_, rpm	21.2 ± 6.2
RR_finish_, rpm	37.1 ± 7.4
CANAC (Q, beats)	5.6 ± 1.07

The average CANAC Q of volunteers in all series in the group as a whole failed to pass the mark of 5.6±1.07 beats (Score “C”).

The degree of correlation of functional indicators (M, HR_M_, GFAR) with CANAC Q for the group as a whole (n=80) was at a very high level (Table 5), which confirmed the effectiveness of using the Coefficient of Anaerobic Capacity (CANAC Q) in assessing the General functional readiness of an athlete.

**Table 5.** The degree of correlation of functional indicators with CANAC Q.

Functional indicators	Group as a whole (n=80)	Degree of correlation with CANAC Q
M, %	67.2 ± 7.2	r = 0.241; p < 0.01
HRM, bpm	137.5 ± 17.1	r = 0.237; p < 0.01
GFAR, units	2.55 ± 0.63	r = 0.406; p < 0.01

## Conclusions

Thus, the results of the study showed a very low average level of the General Functional Athlete Readiness among the volunteers of the university sports club when using the sports standard “R-Engine” (GFAR=2.55±0.63 units with the required level of more than 3.7 units). Moreover, the same result was obtained using a new sports indicator − the Coefficient of Anaerobic Capacity (CANAC Q), which was 5.6 ± 1.07 beats with the required level of more than 10.1 beats.

The fundamental idea of CANAC Q is to calculate the ratio of the increase in heart rate (HR) to the increase in respiratory rate (RR) during intensive physical activity: the lower RR to reach maximum heart rate, the higher the anaerobic capacity of athletes will be. CANAC Q is measured in “beats” of the heart and is recorded very accurately using the method of transthoracic electrical impedance rheography (TEIRG). For this reason, as a promising sports PSM-system, CANAC Q can replace the methods for determining the athlete functional readiness by blood lactate concentration and maximum oxygen consumption.
